# Supplementary material for: Maturational networks of human fetal brain activity reveal emerging connectivity patterns prior to ex-utero exposure
Source: Commun Biol. 2023 Jun 22;6:661. doi: 10.1038/s42003-023-04969-x (PMC10287667; doi:10.1038/s42003-023-04969-x)
Supplement: Supplementary file 3 — Reporting Summary [file 42003_2023_4969_MOESM3_ESM.pdf]

Corresponding author(s): V Karolis
Last updated by author(s): May 5, 2023

Reporting Summary

Nature Portfolio wishes to improve the reproducibility of the work that we publish. This form provides structure for consistency and transparency in reporting. For further information on Nature Portfolio policies, see our Editorial Policies and the Editorial Policy Checklist.

Statistics

For all statistical analyses, confirm that the following items are present in the figure legend, table legend, main text, or Methods section.

n/a	Confirmed	
☐	☒	The exact sample size (*n*) for each experimental group/condition, given as a discrete number and unit of measurement
☒	☐	A statement on whether measurements were taken from distinct samples or whether the same sample was measured repeatedly
☐	☒	The statistical test(s) used AND whether they are one- or two-sided<br>*Only common tests should be described solely by name; describe more complex techniques in the Methods section.*
☐	☒	A description of all covariates tested
☒	☐	A description of any assumptions or corrections, such as tests of normality and adjustment for multiple comparisons
☐	☒	A full description of the statistical parameters including central tendency (e.g. means) or other basic estimates (e.g. regression coefficient) AND variation (e.g. standard deviation) or associated estimates of uncertainty (e.g. confidence intervals)
☐	☒	For null hypothesis testing, the test statistic (e.g. *F*, *t*, *r*) with confidence intervals, effect sizes, degrees of freedom and *P* value noted<br>*Give P values as exact values whenever suitable.*
☒	☐	For Bayesian analysis, information on the choice of priors and Markov chain Monte Carlo settings
☒	☐	For hierarchical and complex designs, identification of the appropriate level for tests and full reporting of outcomes
☒	☐	Estimates of effect sizes (e.g. Cohen's *d*, Pearson's *r*), indicating how they were calculated

Our web collection on statistics for biologists contains articles on many of the points above.

Software and code

Policy information about availability of computer code

Data collection	N/A
Data analysis	Independent component analysis was performed using FSL 6.0.4. Scripts for derivation of matnets is available at: https://gin.g-node.org/slavakarolis/matnet_paper.

For manuscripts utilizing custom algorithms or software that are central to the research but not yet described in published literature, software must be made available to editors and reviewers. We strongly encourage code deposition in a community repository (e.g. GitHub). See the Nature Portfolio guidelines for submitting code & software for further information.

Data

Policy information about availability of data

All manuscripts must include a data availability statement. This statement should provide the following information, where applicable:

- Accession codes, unique identifiers, or web links for publicly available datasets
- A description of any restrictions on data availability
- For clinical datasets or third party data, please ensure that the statement adheres to our policy

The minimum dataset that contain input files necessary to reproduce the results of group-level analyses reported in the manuscript are available at https://gin.g-node.org/slavakarolis/matnet_paper. Source and preprocessed individual data, with recent improvements implemented during ongoing pipeline development, will be made available in the forthcoming release of the dHCP fetal cohort data (anticipated date of release is May 2023).

Human research participants

Policy information about studies involving human research participants and Sex and Gender in Research.

Reporting on sex and gender	Information on the participants' gender is provided in the manuscript. The presence of potential sex differences are outside the scope of the study and not considered to be important for its conclusions
Population characteristics	Fetuses older than 25 weeks of gestation were included in the study. All fetal brain images were reported by a neuroradiologist as showing appropriate appearances for their gestational age with no acquired lesions or congenital malformations of clinical significance.
Recruitment	Participants were prospectively recruited as part of the developing Human Connectome Project, a cross-sectional Open Science initiative.
Ethics oversight	UK National Research Ethics Authority

Note that full information on the approval of the study protocol must also be provided in the manuscript.

Field-specific reporting

Please select the one below that is the best fit for your research. If you are not sure, read the appropriate sections before making your selection.

☒ Life sciences ☐ Behavioural & social sciences ☐ Ecological, evolutionary & environmental sciences

For a reference copy of the document with all sections, see nature.com/documents/nr-reporting-summary-flat.pdf

Life sciences study design

All studies must disclose on these points even when the disclosure is negative.

Sample size	Includes all available and reconstructed data from fetall dHCP cohort at the time of the study initiation.
Data exclusions	Data from 7 fetuses were excluded as they did not pass visual quality assessment due to excessive motion and failure in image reconstruction
Replication	In order to test robustness of the proposed method, its performance was tested in the split-two samples of in-utero data, with results reported in Supplementary Materials, showing a good agreement between the results as well with the result derived from the whole sample. In addition, it was evaluated in an ex-utero sample showing a good agreement with the results derived using an alternative method.
Randomization	N/A. The study utilises an opportunistic sample
Blinding	N/A. No group allocation was needed. The analysis uses age-related changes between subjects as a main contrast.

Reporting for specific materials, systems and methods

We require information from authors about some types of materials, experimental systems and methods used in many studies. Here, indicate whether each material, system or method listed is relevant to your study. If you are not sure if a list item applies to your research, read the appropriate section before selecting a response.

Materials & experimental systems

n/a	Involved in the study	
☒	☐	Antibodies
☒	☐	Eukaryotic cell lines
☒	☐	Palaeontology and archaeology
☒	☐	Animals and other organisms
☒	☐	Clinical data
☒	☐	Dual use research of concern

Methods

n/a	Involved in the study	
☒	☐	ChIP-seq
☒	☐	Flow cytometry
☐	☒	MRI-based neuroimaging

March 2021

Magnetic resonance imaging

Experimental design

Design type	In-utero resting state
Design specifications	N/A
Behavioral performance measures	N/A

Acquisition

Imaging type(s)	fMRI, T2-weighted
Field strength	3T
Sequence & imaging parameters	FMRI: gradient-echo single-shot EPI (TR/TE = 2200/60) sequence consisting of 350 volumes of 48 slices each, slice grid 144 x 144, isotropic resolution = 2.2 mm, multi-band factor = 3 and SENSE factor = 1.4. T2-weighted: TSE
Area of acquisition	Whole brain

Diffusion MRI ☐ Used ☒ Not used

Preprocessing

Preprocessing software	methods developed in-house for fetal brain reconstruction and processing.
Normalization	rigid transformation between subject fMRI and subject T2-weighted using FLS FLIRT and non-linear between subject T2-weighted and template using ANTs
Normalization template	Fetal template available at: https://brain-development.org/brain-atlases/fetal-brain-atlases/
Noise and artifact removal	Utilises both motion parameters and tissue signals, as well as high-path filtering for trend removal
Volume censoring	Rejects outliers based on the signal difference of each volume from a mean volume.

Statistical modeling & inference

Model type and settings	linear regression
Effect(s) tested	Age

Specify type of analysis: ☐ Whole brain ☐ ROI-based ☒ Both

Anatomical location(s)	Where applicable, the ROIs were determined by applying statistical thresholding (Z>3) based on the age related changes in inter-hemispheric connectivity or (in a separate analysis) data-driven parcellation using k-means clustering
Statistic type for inference (See Eklund et al. 2016)	N/A
Correction	N/A

Models & analysis

n/a	Involved in the study
☒	☐ Functional and/or effective connectivity
☒	☐ Graph analysis
☐	☒ Multivariate modeling or predictive analysis

Multivariate modeling and predictive analysis	Independent component analysis implemented as FSL melodic
